# Barriers and Facilitators to the Implementation of Digital Health Services for People With Musculoskeletal Conditions in the Primary Health Care Setting: Systematic Review

**DOI:** 10.2196/49868

**Published:** 2024-08-27

**Authors:** Mark Leendert van Tilburg, Ivar Spin, Martijn F Pisters, J Bart Staal, Raymond WJG Ostelo, Miriam van der Velde, Cindy Veenhof, Corelien JJ Kloek

**Affiliations:** 1 Innovation of Movement Care Research Group Research Centre for Healthy and Sustainable Living HU University of Applied Sciences Utrecht Utrecht Netherlands; 2 Department of Rehabilitation, Physiotherapy Science and Sports UMC Utrecht Brain Center, University Medical Center Utrecht Utrecht University Utrecht Netherlands; 3 Center for Physical Therapy Research and Innovation in Primary Care Julius Health Care Centers Utrecht Netherlands; 4 Research Group Empowering Healthy Behaviour Department of Health Innovations and Technology Fontys University of Applied Sciences Eindhoven Netherlands; 5 Musculoskeletal Rehabilitation Research Group HAN University of Applied Sciences Radboud University Medical Centre Nijmegen Netherlands; 6 Radboud Institute for Health Sciences IQ Healthcare Radboud University Medical Center Nijmegen Netherlands; 7 Department of Health Sciences Faculty of Science VU University, Amsterdam Movement Sciences Research Institute Amsterdam Netherlands; 8 Department of Epidemiology and Data Science Amsterdam University Medical Center Location Vrije Universiteit, Amsterdam Movement Sciences Research Institute Amsterdam Netherlands

**Keywords:** eHealth, primary health care, musculoskeletal problems, implementation science, systematic review, mobile phone

## Abstract

**Background:**

In recent years, the effectiveness and cost-effectiveness of digital health services for people with musculoskeletal conditions have increasingly been studied and show potential. Despite the potential of digital health services, their use in primary care is lagging. A thorough implementation is needed, including the development of implementation strategies that potentially improve the use of digital health services in primary care. The first step in designing implementation strategies that fit the local context is to gain insight into determinants that influence implementation for patients and health care professionals. Until now, no systematic overview has existed of barriers and facilitators influencing the implementation of digital health services for people with musculoskeletal conditions in the primary health care setting.

**Objective:**

This systematic literature review aims to identify barriers and facilitators to the implementation of digital health services for people with musculoskeletal conditions in the primary health care setting.

**Methods:**

PubMed, Embase, and CINAHL were searched for eligible qualitative and mixed methods studies up to March 2024. Methodological quality of the qualitative component of the included studies was assessed with the Mixed Methods Appraisal Tool. A framework synthesis of barriers and facilitators to implementation was conducted using the Consolidated Framework for Implementation Research (CFIR). All identified CFIR constructs were given a reliability rating (high, medium, or low) to assess the consistency of reporting across each construct.

**Results:**

Overall, 35 studies were included in the qualitative synthesis. Methodological quality was high in 34 studies and medium in 1 study. Barriers (–) of and facilitators (+) to implementation were identified in all 5 CFIR domains: “digital health characteristics” (ie, commercial neutral [+], privacy and safety [–], specificity [+], and good usability [+]), “outer setting” (ie, acceptance by stakeholders [+], lack of health care guidelines [–], and external financial incentives [–]), “inner setting” (ie, change of treatment routines [+ and –], information incongruence (–), and support from colleagues [+]), “characteristics of the healthcare professionals” (ie, health care professionals’ acceptance [+ and –] and job satisfaction [+ and –]), and the “implementation process” (involvement [+] and justification and delegation [–]). All identified constructs and subconstructs of the CFIR had a high reliability rating. Some identified determinants that influence implementation may be facilitators in certain cases, whereas in others, they may be barriers.

**Conclusions:**

Barriers and facilitators were identified across all 5 CFIR domains, suggesting that the implementation process can be complex and requires implementation strategies across all CFIR domains. Stakeholders, including digital health intervention developers, health care professionals, health care organizations, health policy makers, health care funders, and researchers, can consider the identified barriers and facilitators to design tailored implementation strategies after prioritization has been carried out in their local context.

## Introduction

### Background

Approximately 1.71 billion people experience musculoskeletal conditions, which are a major contributor to health care problems worldwide [1]. Worldwide population growth and aging will increase the burden of musculoskeletal conditions on health care in the upcoming decades [2,3]. Therefore, prevention, early detection, and optimal treatment of musculoskeletal conditions, which comprise one of the largest patient groups in primary care, become increasingly important [4-6]. However, patients experience barriers that decrease access to primary care services, such as geographic and transport-related barriers, lack of health insurance, no after-hours access, and a shortage of primary health care professionals [7,8]. A potential solution to optimize prevention and treatment of musculoskeletal conditions, reduce the burden of musculoskeletal conditions on health care, and improve accessibility in primary care is the use of digital health services.

Digital health is an umbrella term encompassing eHealth and mobile health, which are defined as the use of information and communications technology in support of health and health-related fields and the use of mobile wireless technologies for health [9]. Examples are video consultations between a health care professional and patient and the integration of apps within primary care treatment. There are several potential benefits to digital health services, such as improved cost-effectiveness, more information about the health status of the patient, better communication between patients and health care professionals, and more accessibility for patients [10,11]. Previous research supports the effectiveness of digital health services in reducing pain and improving functional disability, catastrophizing, coping ability, and self-efficacy [12,13].

Despite the benefits of digital health services, their use for musculoskeletal conditions in primary care is lagging. Therefore, a thorough implementation is needed, including the development of implementation strategies that potentially improve the use of digital health services for patients with musculoskeletal conditions in primary care. Important stakeholders for designing these implementation strategies are eHealth developers, health care professionals, health care organizations, health policy makers, health care funders, and researchers. The first step to design implementation strategies for local contexts is to perform a determinant analysis in a more specific context to gain insight into determinants that influence implementation from the perspective of patients and health care professionals. Several studies have identified barriers and facilitators to the implementation of digital health services in other settings and populations [14-17]. Some of these barriers for patients or health care professionals in these settings are workflow, resistance to change, costs, reimbursement, intervention design, and digital literacy. However, it remains unclear which barriers and facilitators are applicable for patients with musculoskeletal conditions in the primary health care setting and what the overarching narrative is for this patient population and setting. A generic overview of barriers and facilitators within this more specific context, which is the aim of this systematic review, is useful as a first step for a thorough implementation. A prioritization of these barriers and facilitators for various local contexts, that is, a specific primary care physiotherapy practice, would be the next step to design fitting implementation strategies for the local context [18].

A practical theory-based framework to guide for systematical assessment of barriers and facilitators that influence implementation is the Consolidated Framework for Implementation Research (CFIR) [18]. The CFIR consolidates implementation determinants from a broad array of implementation theories and is composed of 5 domains (intervention [digital health service] characteristics, outer setting, inner setting, characteristics of individuals [health care professionals], and the implementation process), and it provides a systematic way of identifying constructs that have been associated with effective implementation. The use of a framework such as CFIR to structure the overview of barriers and facilitators allows stakeholders undertaking implementation activities to focus on barriers and facilitators that are of most interest to them more easily and design implementation strategies that are specific to their local context [19].

### Objectives

No systematic overview of barriers and facilitators influencing the implementation of digital health services for people with musculoskeletal conditions in the primary health care setting exists to support these stakeholders in designing fitting implementation strategies. Therefore, the aim of this systematic literature review was to identify barriers and facilitators to the implementation of digital health for people with musculoskeletal conditions in the primary health care setting.

## Methods

This systematic literature review of qualitative data from qualitative and mixed methods articles is reported following the enhancing transparency in reporting the synthesis of qualitative research statement [20]. Exclusively incorporating qualitative evidence in this systematic review enables a nuanced exploration of the multifaceted factors influencing implementation, providing diverse perspectives and in-depth insights from both patients and health care providers.

### Search Strategy

The electronic databases PubMed, Embase, and CINAHL were searched to seek all available studies up to March 2024. The complete search strategy can be found in Multimedia Appendix 1. Inclusion and exclusion criteria are presented in Textbox 1.

**Inclusion and exclusion criteria.**

**Inclusion criteria**

- Domain: adults (aged ≥18 years); musculoskeletal conditions (eg, low back pain, osteoarthritis, and total knee replacement); primary health care setting (eg, general practice and physiotherapy practice)
- Determinant: the health care professional (eg, general practitioners, physiotherapists, and occupational therapists) has provided digital health (eg, synchronous patient-therapist interactions through telephone or video consultations and asynchronous physical exercise training, coaching, and monitoring using web applications, wearables, and platforms) more than once during an intervention
- Outcome: data on barriers or facilitators to the implementation of digital health services that fit into one of the Consolidated Framework for Implementation Research domains; data of patients or health care professionals
- Article type: qualitative and mixed methods designs; full text in English is available

**Exclusion criteria**

- Determinant: web-based training programs for health care professionals
- Article type: articles with no qualitative component, such as a quantitative survey only

### Selection of Studies

**Figure 1:**
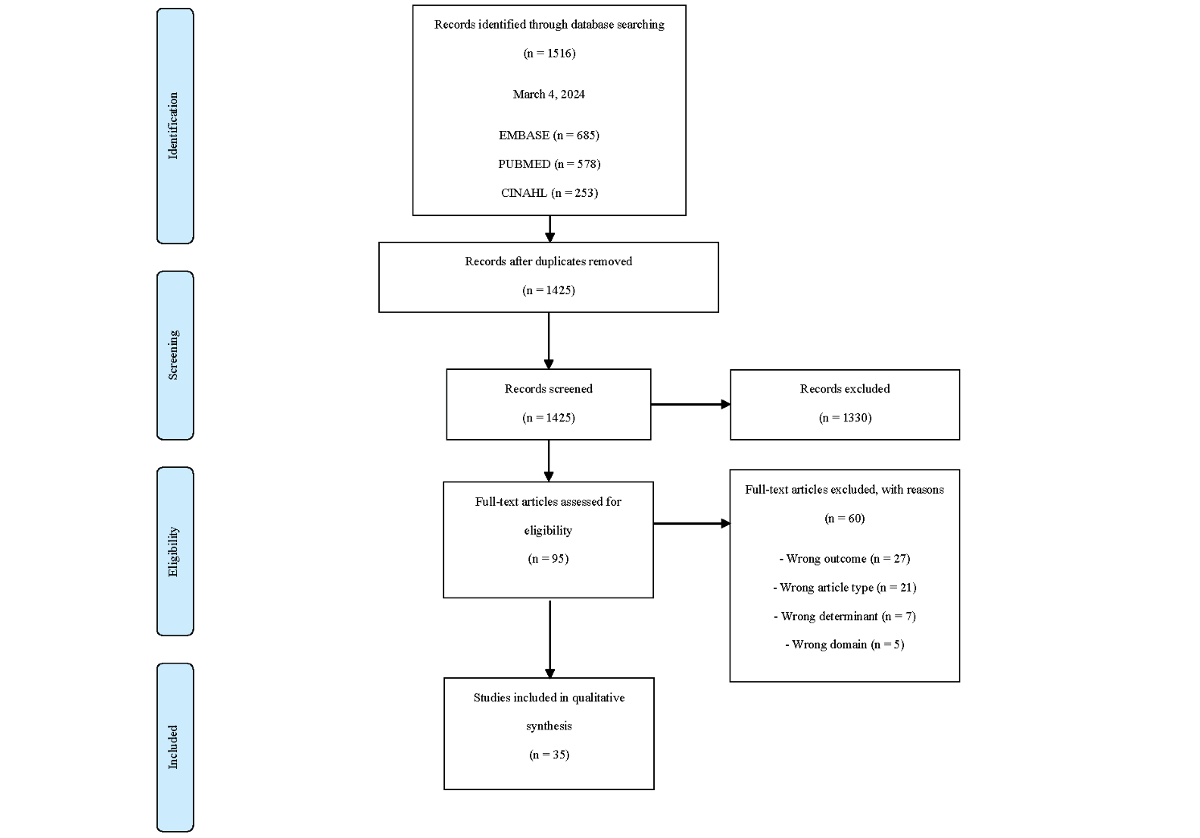
PRISMA (Preferred Reporting Items for Systematic Reviews and Meta-Analyses) flow diagram.

The web-based screening tool Rayyan was used for the selection of studies [21]. A total of 3 reviewers (MLvT, IS, and MvdV) conducted the inclusion of eligible articles. Articles were screened independently by 2 reviewers for eligibility based on title and abstract. When an article was potentially eligible for inclusion, a full paper copy of the report was obtained and screened independently by 2 reviewers. Disagreements between the reviewers regarding an article’s eligibility were resolved by discussion until consensus was reached. In case of disagreement, a fourth reviewer (CJJK) was consulted. In addition, reference tracking was performed in all included articles. The reasons for exclusion were recorded (Figure 1) [22].

### Data Extraction and Management

The reviewers extracted the following data using a standardized extraction form: first author, country, year of publication, aim, design and method of data collection and methods of analysis, sample, description of digital health service, and data on barriers or facilitators to the implementation of digital health services reported in the Results section.

### Assessment of Methodological Quality

In total, 3 reviewers (MLvT, IS, and MvdV) independently assessed the methodological quality of the qualitative component of the included articles. The Mixed Methods Appraisal Tool (MMAT) was used to appraise studies for this review [23]. It is a 5-item tool designed to appraise the methodological quality of 5 categories of studies, including qualitative and mixed methods studies. The MMAT has established content validity and has been piloted across the mentioned methodologies [24]. The MMAT encompasses 2 initial screening questions: “Are there clear research questions?” and “Do the collected data allow to address the research questions?” Methodological quality assessment was only performed if “yes” could be answered for both screening questions. A detailed presentation of the individual ratings of each MMAT criterion was provided to inform the quality of the included studies. Overall sum scores were calculated based on the quality of the qualitative component only and presented as number of stars (*), with 0 and 1 star indicating low quality, 2 and 3 stars indicating medium quality, and 4 and 5 stars indicating high quality [25]. These cutoff values were determined by 2 reviewers (MLvT and IS), as the MMAT subscribes, and are arbitrary but useful for transparent data syntheses. Disagreements were resolved in a consensus meeting between the raters. When there was any disagreement, a fourth reviewer (CJJK) could be consulted but was not necessary. As the aim was to describe and synthesize a body of qualitative literature and not determine an effect size, the quality assessment was only included to inform the overall quality of the included articles and to determine the reliability rating.

### Data Synthesis

A framework synthesis was performed, with secondary thematic analysis of the results section of the included articles. To synthesize the findings, the CFIR was used, using the 2009 version because the analysis began before the 2022 update [18]. Initially, MLvT and IS used an open coding process to identify barriers and facilitators to implementation and allocated them to the most fitting CFIR construct or subconstruct using a coding manual from the CFIR [26]. During the axial coding process, these open codes were organized into thematic categories representing barriers and facilitators to implementation. MLvT conducted the axial coding, which was reviewed by IS and CJJK on an iterative basis. As the thematic analysis progressed, recurring themes identified across the included studies informed the development of a comprehensive narrative for the generic overview of barriers and facilitators to implementation.

Next, all identified CFIR constructs or subconstructs were given (MLvT, IS, and MvdV) a reliability rating to review the consistency of reporting across each construct and the quality of the studies that identified them, which was also reported in another systematic review on barriers and facilitators in another context and aims to indicate confidence in the findings [27]. All disagreements were resolved through discussion until consensus was reached. Three levels of reliability were distinguished: (1) high reliability (the construct is consistently supported by >1 study of medium quality and 1 study of high quality or the construct is supported by at least 2 studies of high quality based on the MMAT); (2) medium reliability (the construct is supported by >1 study of medium quality or the construct is identified on the basis of at least 1 high-quality study based on the MMAT); and (3) low reliability (the construct is supported only by studies of low quality or single studies of medium quality based on the MMAT).

## Results

### Study Selection

The literature search resulted in a total of 1516 articles found in the Embase, PubMed, and CINAHL databases. After removing duplicates, 1425 articles were screened based on title and abstract. This resulted in 95 studies that were screened full text, after which studies were excluded on outcome (n=27, 28%), article type (n=21, 22%), determinant (n=7, 7%), and domain (n=5, 5%). In 2 cases of initial disagreement between reviewers, a fourth reviewer (CJJK) was consulted. Finally, 35 studies were included in the qualitative synthesis [28-62]. No additional studies were found through reference checking. The study selection procedure is presented in Figure 1.

### Study Characteristics

Characteristics of the included studies are presented in Table 1. Individual articles are ordered alphabetically within all presented tables. All included articles were published between 2011 and 2024. A total of 10 articles originated from Australia [31,38,40,44,47,48,52,54,57,60]; 9 articles originated from the Netherlands [28-30,35,39,43,45,46,62]; 4 articles originated from Canada [34,37,41,50]; 3 articles originated from the United Kingdom [51,53,59]; 2 articles originated from Brazil [36,58], Sweden [32,55], and France [42,61]; and 1 article originated from Denmark [56]. The digital health services mentioned in the included articles aimed to facilitate synchronous patient-therapist interactions through telephone or video consultations and to support asynchronous physical exercise training, coaching, and monitoring using web applications and platforms. The participants in the included articles primarily consisted of patients, physiotherapists, and general practitioners but also encompassed occupational therapists, dietitians, psychologists, and a pharmacist. Patients presented with a variety of musculoskeletal conditions, including knee and hip osteoarthritis, knee conditions, chronic nonspecific low back pain, Achilles tendinopathy, traumatic hand injury, anterior cruciate ligament reconstruction, shoulder joint replacement, and total knee replacement.

Information about methodological quality of the studies is presented in Table 2. Almost all qualitative components of the included studies, assessed with the MMAT, were of high methodological quality. In total, 31 articles scored 5 stars, and 3 articles scored 4 stars. Qualitative component of 1 article was of medium methodological quality and scored 3 stars.

**Table 1:** Characteristics of the included studies.

Author, year, country	Aims of the study	Methods^a^	Participants^a^	Digital health service
Aily et al [58], 2020; Brazil	To investigate whether people with knee osteoarthritis would adhere to an exercise therapy program delivered via multiple media To analyze the effects of intervention on pain and function To compare acceptability of the telerehabilitation program by middle-aged and older people involved in the study	Design: mixed methods Data collection: focus group Data analysis: inductive thematic analysis	People with knee osteoarthritis (n=6)	In-person exercise therapy instructions along with a booklet and DVD to take home. Participants also received 6 motivational phone calls throughout the 12-week treatment
Arensman et al [43], 2022; the Netherlands	To investigate patient perspectives on the acceptability, satisfaction, and performance of an app to support home-based exercise following recommendations from a physiotherapist	Design: qualitative Data collection: interviews Data analysis: framework method	People with LBPb (n=9) Women, n=5 (56%) Age (years), minimum-maximum: 20-71	The Physitrack app that allows physiotherapists to create and share personalized exercise programs with patients. The app allows patients to set reminders to perform their exercises, track their adherence, rate pain scores during the exercises, and send direct messages to their physiotherapists
Barton et al [40], 2022; Australia	To explore the experiences and attitudes of people receiving physiotherapy telehealth services for musculoskeletal pain conditions during the COVID-19 pandemic	Design: sequential mixed methods Data collection: semistructured interviews Data analysis: inductive thematic analysis	People with musculoskeletal pain (n=19) Women, n=11 (60%) Age (years), mean 53 (SD 17)	Telehealth care from physiotherapists throughout Australia
Bossen et al [62], 2016; the Netherlands	To develop a blended exercise therapy intervention for people with knee and hip osteoarthritis that matches the values of the users and that can be implemented in the daily routine of physiotherapists To investigate the feasibility through interviews and a pilot study	Design: mixed methods Development phase Data collection: focus group and stakeholder committee Data analysis: summarizing Pilot study Data collection: interviews Data analysis: thematic trend analysis	Development phase Focus group: physiotherapists with extensive experience in the field of osteoarthritis (n=7) Stakeholder committee: people with knee and hip osteoarthritis, the Royal Dutch Society for Physical Therapy, 2 rehabilitation centers, the Dutch arthritis foundation, an eHealth entrepreneur, and a health insurer (n=7) Pilot study: physiotherapists (n=5) and people with osteoarthritis (n=4)	E-Exercise is a 12-week intervention, which combines visits with a physiotherapist and a web-based physical activity intervention. Patients receive 4 face-to-face sessions and are supposed to complete 12 web-based assignments. The website has a portal for both patients and physiotherapists and contains text- and video-based information
Button et al [51], 2018; the United Kingdom	To integrate TRAKc into the physiotherapy outpatient service of 1 National Health Service Health Board and to evaluate patient and physiotherapist use and views of TRAK	Design: mixed methods Data collection: interviews Data analysis: inductive thematic approach	People with knee conditions (n=16) Women, n=10 (63%) Age (years), mean: 39 Physiotherapists (n=15)	TRAK is a web-based intervention for supporting rehabilitation of knee conditions, with a potential to enhance the quality of treatment components, such as health information provision, rehabilitation monitoring, remote support, and personalized exercise progression
Martínez de la Cal et al [49], 2021; Spain	To explore physiotherapists’ opinions of the efficacy, benefits, and disadvantages of implementing a web-based telerehabilitation program in the treatment of chronic nonspecific low back pain To explore the experience of physiotherapists in the management of people with chronic nonspecific LBP	Design: qualitative Data collection: interviews Data analysis: thematic analysis	Physiotherapists (n=19) Women, n=8 (42%) Age (years), mean 39 (SD 8) Professional experience (years), mean 15 (SD 7)	McKenzie Exercise Therapy and electroanalgesia based on telerehabilitation with the help of 10.1 “Quad Core” tablets
Cottrell et al [52], 2017; Australia	To evaluate service provider’s views on Current barriers to patients’ accessing N/OPSCd and MDSe The implementation of telerehabilitation within the N/OPSC and MDS	Design: qualitative Data collection: semistructured interviews Data analysis: template analysis	Service providers (n=26) Woman, n=16 (61%) Physiotherapy, n=15 (58%)	Telerehabilitation: delivery of rehabilitation service at a distance using telecommunications technology
Dehainault et al [42], 2024; France	To explore the content of physical activity for low back pain that general practitioners provide and their opinion about health care smartphone eHealth apps as a support for this advice	Design: qualitative Data collection: semistructured interviews Data analysis: thematic analysis	General practitioners (n=16) Women, n=9 (56%) Age (years), mean (minimum-maximum): 43 (29-64)	Participants were presented with screenshots from the “Mon Coach Dos” and “Activ’Dos” mobile apps, with a standardized explanatory presentation framework. An information sheet about the apps was integrated into the slideshow presenting their description, creator, funding, and data use
Dunphy et al [53], 2017; the United Kingdom	To evaluate the acceptability of TRAK to people following ACLf reconstruction To evaluate the acceptability of TRAK to physiotherapists	Design: qualitative Data collection: semistructured interviews Data analysis: pragmatic thematic analysis	People following ACL reconstruction (n=17) Woman, n=7 (41%) Age (years), mean: 30 Physiotherapists (n=4)	TRAK is a digital intervention developed to support self-management of knee conditions. TRAK provides a platform for individually tailored exercise programs with videos, detailed instructions and progress logs for individual exercises, a health information section, and a contact option that allows a patient to email a physiotherapist for additional support
Egerton et al [54], 2017; Australia	To identify GPs’g perspectives on potential barriers and facilitators to engagement with a new model to support knee osteoarthritis management with remote delivery options	Design: qualitative Data collection: interviews Data analysis: inductive thematic approach	General practitioners (n=11) Woman, n=7 (64%) Age (years), mean (minimum-maximum): 51 (34-67)	The new model for primary care management of knee osteoarthritis includes a multidisciplinary team of health professionals using remote delivery options (primarily telephone) to provide ongoing “care support.” The GP refers the patient to the “care support team” following a brief initial consultation. The “care support team” staff will have skills in health behavior change plus expertise in current best practice for knee osteoarthritis management
Eriksson et al [55], 2011; Sweden	To describe patients’ experiences with home-based physiotherapy via video link after shoulder joint replacement	Design: qualitative Data collection: interviews Data analysis: qualitative content analysis	People after shoulder joint replacement (n=10) Age (years), median (minimum-maximum): 70 (53-85) Woman, n=8 (80%)	A 2-month home-based video physiotherapy program, supervised by an experienced physiotherapist specializing in shoulder problems.
Ezzat et al [38], 2022; Australia	To understand patients’ perceived acceptability of participating in a telehealth-delivered group-based education and exercise therapy program for knee osteoarthritis	Design: qualitative Data collection: semistructured interviews Data analysis: inductive analysis	People with knee osteoarthritis (n=19) Women, n=12 (63%) Age (years), mean (minimum-maximum): 62 (49-72) Using telehealth program, n=11 (58%)	GLA:D^h^ is a physiotherapist-led 8-week program, which includes 2 group education sessions, followed by 12 supervised, neuromuscular exercise therapy sessions. The program is delivered via telehealth or in person
Ezzat et al [44], 2023; Australia	To evaluate the implementation of GLA:D via telehealth in Australia using physiotherapist and patient data and applying the RE-AIM QuESTi framework	Design: convergent mixed methods Data collection: semistructured interviews Data analysis: inductive, reflexive thematic analysis	Physiotherapists (n=23) Women, n=14 (61%)	GLA:D is a physiotherapist-led 6- to 8-week program, which includes 2 to 3 group education sessions, followed by 12 supervised, neuromuscular exercise therapy sessions. The program is delivered via telehealth or in person
Farzad et al [41], 2023; Canada	To explore the perspective and experiences of hand therapists from different countries in providing telerehabilitation to understand the barriers and facilitators that the therapists faced during their web-based interventions in hand therapy	Design: qualitative Data collection: interviews Data analysis: qualitative content analysis	Occupational and physiotherapists (n=14) Women, n=12 (86%) Age (years), mean: 44	Web-based hand therapy interventions
Geraghty et al [59], 2020; the United Kingdom	To explore patients’ experiences of using the SupportBack internet intervention, both with and without physiotherapist telephone support	Design: embedded qualitative Data collection: interviews Data analysis: thematic analysis	People with nonspecific LBP (n=15) Women, n=10 (67%) Age (years), mean 60 (SD 15)	SupportBack is a web-based platform to support patients through a self-tailored, 6-week self-management program. Contents include exercises or a walking program with weekly goals, feedback, and advice. Patients also received 3 telephone calls from an musculoskeletal physiotherapist to provide reassurance, address concerns, problem-solve, and encourage continued engagement with the intervention and physical activity goals
Hasani et al [60], 2021; Australia	To explore the experience of participants and physiotherapists with gym-based exercise interventions for Achilles tendinopathy with weekly telehealth monitoring	Design: embedded qualitative Data collection: interviews, focus group Data analysis: thematic analysis	Interviews People with Achilles tendinopathy (n=8) Age (years), minimum-maximum: 38-54 Focus group Physiotherapists (n=7) Women, n=2 (29%) Age (years), minimum-maximum: 25-44 Professional experience (years), minimum-maximum: 3-22	Gym-based exercise program where the participants performed 4 sets of unilateral isotonic standing and seated calf raise exercises in a Smith machine (both sides, one leg at a time) 3 times per week, over 12 weeks Physiotherapists supervised 1 session per week via videoconference software (Zoom) that was downloaded to the participants’ smartphone
Hinman et al [48], 2017; Australia	To explore the experience of patients and physiotherapists using Skype as a service delivery model for physiotherapist-prescribed exercise management of knee osteoarthritis	Design: embedded qualitative Data collection: interviews Data analysis: thematic analysis	People with knee osteoarthritis (n=12) Women, n=6 (50%) Age (years), mean 62 (SD 7)	Participants were provided 7 internet-based Skype-delivered physical therapy sessions for 3 months, with the main purpose being to prescribe an individualized home-based strengthening program to be undertaken 3 times per week
Hjelmager et al [56], 2019; Denmark	To identify GPs’ barriers and facilitators regarding the use of health information technology in the treatment of people with LBP	Design: qualitative Data collection: interviews Data analysis: inductive thematic analysis	General practitioners (n=8) Women, n=4 (50%) Age (years), minimum-maximum: 41-66	Distribution of health-related information via the internet
Kairy et al [50], 2013; Canada	To explore the perception of people who have undergone a total knee replacement concerning in-home telerehabilitation services	Design: embedded qualitative Data collection: interviews Data analysis: thematic	People after total knee replacement (n=5) Women, n=3 (60%) Age (years), minimum-maximum: 44-72	An in-home telerehabilitation program consisting of twice-a-week physiotherapy sessions for 8 weeks (total 16 sessions) by a videoconferencing system located in the participant’s home
Kelly et al [33], 2022; Ireland	To explore the perceptions of eHealth-mediated supported self-management from the perspective of people with musculoskeletal disorders and physiotherapists	Design: qualitative Data collection: semistructured interviews Data analysis: reflexive thematic analysis	People with musculoskeletal disorders (n=13) Women, n=9 (69%) Age (years), mean (minimum-maximum): 58 (24-77) musculoskeletal physiotherapists (n=13) Women, n=6 (46%) Age (years), mean (minimum-maximum): 35 (26-42)	The use of technological platforms (eg, mobile, computer and tablet) in physiotherapy
Kingston et al [57], 2015; Australia	To explore the experiences of people receiving medical treatment and rehabilitation for a traumatic hand injury	Design: qualitative Data collection: interviews Data collection: inductive interpretive phenomenological approach	People receiving medical treatment and rehabilitation for a traumatic hand injury (n=14) Age (years), minimum-maximum: 24-82	The use of technology, namely, telehealth and the use of the internet
Kloek et al [46], 2020; the Netherlands	To explore the experiences of physiotherapists and identify determinants that facilitate and hinder the use of the blended intervention e-Exercise	Design: embedded mixed methods Data collection: interviews Data analysis: grounded theory methodology	Physiotherapists (n=9) Women, n=3 (33%) Age (years), minimum-maximum: 24-59	The intervention consists of about 5 physiotherapy sessions in combination with a web-based application (E-Exercise). The web-based application contains a tailored 12-week behavioral graded activity program, videos with strength and mobility exercises, and videos and texts with information about osteoarthritis-related topics.
Lamper et al [39], 2021; the Netherlands	To explore the feasibility of the eCoach Pain for people with chronic musculoskeletal pain and primary health care professionals	Design: mixed methods Data collection: focus group and interviews Data analysis: thematic analysis	People with chronic musculoskeletal pain (n=11) Women, n=8 (73%) Age (years), mean 60 (IQR 2) Primary health care professional (n=6) Women, n=4 (67%)	The eCoach Pain is an electronic coach to facilitate pain rehabilitation. It supports the provision of integrated rehabilitation care with a shared biopsychosocial vision on health. Both patients and primary health care professionals use the eCoach Pain. It comprises a measurement tool for assessing complexity of the pain problem, diaries, pain education sessions, monitoring options, and a chat function
Lawford et al [47], 2019; Australia	To qualitatively explore whether physiotherapists’ perceptions about telephone-delivered exercise therapy for people with knee osteoarthritis shifted once they had delivered exercise management advice to people with knee osteoarthritis over the telephone	Design: embedded qualitative Data collection: pre- and postinterviews Data analysis: thematic analysis	Physiotherapists (n=8) Women, n=4 (50%) Professional experience (years), mean 14 (SD 8)	The patients received 5 to 10 telephone consultations over a 6-month period. Physiotherapists devised goals and an action plan for each patient that involved both a structured home exercise program and a physical activity plan. Patients also had access to a study website containing video demonstrations of each exercise
Van der Meer et al [29], 2022; the Netherlands	To assess the needs, facilitators, and barriers of the use of an eHealth application from the perspective of both orofacial physiotherapists and people with TMDj	Design: qualitative Data collection: interviews Data analysis: thematic analysis	Orofacial physical therapists (n=11) People with TMD (n=9)	eHealth included in the health care process of people with TMD
Östlind et al [32], 2022; Sweden	To explore the experiences of using a wearable activity tracker to monitor physical activity and the general perceptions of digital support in osteoarthritis care among people of working age with hip and knee osteoarthritis	Design: qualitative Data collection: focus groups Data analysis: content analysis	Working individuals with hip or knee osteoarthritis (n=18) Women, n=13 (72%) Age (years), mean 58 (SD 6)	A wearable activity tracker (Fitbit Flex 2) in combination with the Fitbit app for 12 weeks. The participants were asked to monitor their activity daily, and they received automatic feedback from the app
Palazzo et al [61], 2016; France	To assess views of people with chronic LBP concerning barriers to home-based exercise program adherence and to record expectations regarding new technologies	Design: qualitative Data collection: semistructured interviews Data analysis: iterative inductive analysis	People with chronic LBP (n=29) Women, n=17 (59%) Age (years), mean (minimum-maximum): 54 (24-85)	The use of new technologies to decrease the burden of home-based exercise programs in chronic LBP
Passalent et al [37], 2022; Canada	To understand patient perspectives of the importance of physical activity in the management of axial spondyloarthritis To describe motivators and barriers associated with adherence to physical activity in people with axial spondyloarthritis To explore the role of eHealth technology in facilitating physical activity in people with axial spondyloarthritis	Design: qualitative Data collection: semistructured interviews Data analysis: thematic analysis	People with axial spondyloarthritis (n=12) Women, n=2 (17%) Age (years), mean 46 (SD 13)	Technology for encouraging physical activity
Pereira et al [36], 2023; Brazil	To explore beliefs and expectations of individuals with fibromyalgia about physical exercises delivered through telerehabilitation	Design: qualitative Data collection: semistructured interviews Data analysis: inductive approach	People with fibromyalgia (n=30) Women, n=30 (100%) Age (years), mean 45 (SD 11)	Physical therapy by telerehabilitation
Petrozzi et al [31], 2021; Australia	To understand the experiences of people with LBP with the Mind Your Back Trial	Design: qualitative Data collection: interviews Data analysis: thematic analysis	People with LBP (n=25) Women, n=12 (48%) Age (years), mean 53 (SD 13)	Physical treatments combined with an internet-delivered psychosocial program called MoodGYM
Poolman et al [35], 2024; the Netherlands	To gain insights in how participants experienced the Back2Action intervention	Design: qualitative Data collection: semistructured interviews Data analysis: thematic analysis	People with nonspecific LBP and neck pain (n=11) Women, n=6 (55%) Age (years), median 48 (IQR 25-44)	Back2Action is a newly developed biopsychosocial-blended intervention consisting of in-person physiotherapy sessions blended with psychologically informed digital health. The digital part of the intervention incorporates pain education and behavioral activation
Renard et al [34], 2022; Canada	To evaluate the acceptability of 2 remote follow‐up modalities (telephone and teleconsultation) for patients waiting for public rehabilitation services	Design: qualitative Data collection: semistructured interviews Data analysis: content analysis	People with nonurgent musculoskeletal conditions (n=10) Women, n=7 (70%) Age (years), mean 49 (SD 14)	Teleconsultation follow‐ups
Van Tilburg et al [45], 2022; the Netherlands	To investigate the feasibility of the e-Exercise LBP prototype for patients and physiotherapists	Design: embedded mixed methods Data collection: interviews Data analysis: thematic analysis	People with LBP (n=7) Women, n=4 (57%) Age (years), mean 45 (SD 11) Physiotherapists (n=7) Women, n=4 (57%) Age (years), mean 37 (SD 12)	The patients received a stratified blended intervention, whereby a prognostic stratification tool, a web-based application (e-Exercise), and face-to-face physiotherapy sessions are integrated within physiotherapy treatment to create an optimal combination
Van Tilburg et al [30], 2023; the Netherlands	To develop physiotherapy-specific matched treatment options as part of a new Stratified Blended Physiotherapy approach for people with neck and shoulder conditions To investigate feasibility of the Stratified Blended Physiotherapy approach for people with neck and shoulder conditions	Design: 2-phase mixed methods Phase 1 Data collection: focus groups Data analysis: thematic analysis Phase 2 Data collection: semstructured interviews Data analysis: thematic analysis	Phase 1 Stakeholders with expertise in the field of eHealth, stratified care and neck and shoulder conditions (n=17) Phase 2 Primary care physiotherapists (n=8) People with neck and shoulder conditions (n=13)	A blended physiotherapy treatment (e-Exercise) for people with neck and shoulder conditions in which a smartphone app with personalized information, exercises, and physical activity modules was an integral part of physiotherapy treatment
De Vries et al [28], 2017; the Netherlands	To explore what patient-, intervention-, and environment-related determinants are determinants of adherence to the web-based component of e-Exercise	Design: embedded mixed methods Data collection: interviews Data analysis: grounded theory methodology	People with hip and knee osteoarthritis (n=10) Women, n=7 (70%) Age (years), mean (minimum-maximum): 60 (51-79)	The web-based component of e-Exercise consists of a 12-week incremental physical activity program based on graded activity, strength and stability exercises, and information on osteoarthritis-related themes. The offline component consists of up to 5 face-to-face physiotherapy sessions

^a^For mixed methods designs, only the data collection, data analysis, and participants from the qualitative component are described.

^b^LBP: low back pain.

^c^TRAK: Taxonomy for the Rehabilitation of Knee Conditions.

^d^N/OPSC: Neurosurgical and Orthopaedic Physiotherapy Screening Clinic.

^e^MDS: multidisciplinary service.

^f^ACL: anterior cruciate ligament.

^g^GP: general practitioner.

^h^GLA:D: Good Life with Osteoarthritis in Denmark.

^i^RE-AIM QuEST: Reach, Effectiveness, Adoption, Implementation, and Maintenance Qualitative Evaluation for Systematic Translation.

^j^TMD: temporomandibular disorder.

**Table 2:** Methodological quality of the included studies.

Study	Criteria from the Mixed Methods Appraisal Tool: qualitative studies						Total number of stars (based on the qualitative component)
	1.1^a^	1.2^b^	1.3^c^	1.4^d^	1.5^e^		
Aily et al [58], 2020	0^f^	1^g^	1	1	1	***	
Arensman et al [43], 2022	1	1	1	1	1	*****	
Barton et al [40], 2022	1	1	1	1	1	*****	
Bossen et al [62], 2016	1	1	1	0	1	****	
Button et al [51], 2018	1	1	1	1	1	*****	
Martínez de la Cal et al [49], 2021	1	1	1	1	1	*****	
Cottrell et al [52], 2017	1	1	1	1	1	*****	
Dehainault et al [42], 2024	1	1	1	1	1	*****	
Dunphy et al [53], 2017	1	1	1	1	1	*****	
Egerton et al [54]	1	1	1	1	1	*****	
Eriksson et al [55], 2011	1	1	1	0	1	****	
Ezzat et al [38], 2022	1	1	1	1	1	*****	
Ezzat et al [44], 2023	1	1	1	1	1	*****	
Farzad et al [41], 2023	1	1	1	1	1	*****	
Geraghty et al [59], 2020	1	1	1	1	1	*****	
Hasani et al [60], 2021	1	1	1	1	1	*****	
Hinman et al [48], 2017	1	1	1	1	1	*****	
Hjelmager et al [56], 2019	1	1	1	1	1	*****	
Kairy et al [50], 2013	1	1	1	1	1	*****	
Kelly et al [33], 2022	1	1	1	1	1	*****	
Kingston et al [57], 2015	1	1	1	1	1	*****	
Kloek et al [46], 2020	1	1	1	1	1	*****	
Lamper et al [39], 2021	1	1	1	1	1	*****	
Lawford et al [47], 2019	1	1	1	1	1	*****	
van der Meer et al [29], 2022	1	1	1	1	1	*****	
Östlind et al [32], 2022	1	1	1	1	1	*****	
Palazzo et al [61], 2016	1	1	1	1	1	*****	
Passalent et al [37], 2022	1	1	1	1	1	*****	
Pereira et al [36], 2023	1	1	1	1	1	*****	
Petrozzi et al [31], 2021	1	1	1	1	1	*****	
Poolman et al [35], 2024	1	1	1	1	1	*****	
Renard et al [34], 2022	1	1	1	1	1	*****	
van Tilburg et al [45], 2022	1	1	1	1	1	*****	
van Tilburg et al [30], 2023	1	1	1	1	1	*****	
De Vries et al [28], 2017	1	1	1	0	1	****	

^a^1.1=Is the qualitative approach appropriate to answer the research question?

^b^1.2=Are the qualitative data collection methods adequate to address the research question?

^c^1.3=Are the findings adequately derived from the data?

^d^1.4=Is the interpretation of results sufficiently substantiated by data?

^e^1.5=Is there coherence between qualitative data sources, collection, analysis, and interpretation?

^f^0=no.

^g^1=yes.

### Barriers and Facilitators by CFIR

#### Overview

An overview of CFIR constructs or subconstructs influencing implementation of digital health services for patients with musculoskeletal conditions in the primary health care setting, with the sources and reliability rating, is presented in Table 3. An overview of the data synthesis supported by illustrative quotes, is presented in Table 4.

**Table 3:** Constructs that influence implementation of digital health.

CFIR^a^ domain, construct, and subconstruct				Studies		Reliability
**Innovation characteristics**						
	Innovation source		[42,53,56]		High	
	Relative advantage		[28-45,47-50,52-57,59-62]		High	
	Adaptability		[28-34,39,41-46,48,50,52-54,56,57,59,61,62]		High	
	Complexity		[28-30,37,40,43-45,48,50,51,53,55,56,59,60]		High	
	Design quality and packaging		[29,33,34,40,45,48,50,53,55,56,59,61,62]		High	
	Cost		[29,34,40,41,46,47,52,54,55,62]		High	
**Outer setting**						
	Patient needs and resources		[28,31,33-36,38,41,42,44-48,50-53,55,57-62]		High	
	External policy and incentives		[29,31,32,41,44,46,62]		High	
**Inner setting**						
	Networks and communications		[39,52,54]		High	
	**Implementation climate**					
		Tension for change	[29,44,49,52,54,56,57]		High	
		Compatibility	[29,30,39,44,46-48,51,52,54]		High	
		Learning climate	[45,46]		High	
	**Readiness for implementation**					
		Available resources	[28,30,32,33,36,38,40,41,43-45,48,49,51-53,56,60,61]		High	
		Access to knowledge and information	[45-47,51,52,60]		High	
**Characteristics of individuals**						
	Knowledge and beliefs about the intervention		[29,33,42,44-49,51-54,56,60,62]		High	
**Process**						
	**Engaging**					
		Opinion leaders	[56]		Medium	
		Key stakeholders (health care professional)	[50,52,54,60]		High	
	Executing		[60]		Medium	

^a^CFIR: Consolidated Framework for Implementation Research.

**Table 4:** Overview of the data synthesis supported by illustrative quotes.

CFIR^a^ domain, construct, and subconstruct					Barriers (–) and facilitators (+) with illustrative quotes
**Digital health service characteristics**					
	Innovation source			Commercially neutral (+) “Some could not see themselves ‘offering patients something done by a lab’ since it was unlikely the labs were ‘doing this for philanthropic reasons.’” [General practitioner] [42] Link with an institution with a good image (+) “I think it’s really good because I have heard that other hospitals... doesn’t have a programme that is as good as yours and physios that look after you.” [Patient] [53]	
	Relative advantage			Adherence (+) “In my busy life, the reminders motivated me to take some time to get it done.” [Patient] [43] Self-management (+) “I think it did take away from that expectation of manual therapy. I know when people come into the clinic and they’re coming in for a similar issue...because you’re in the room with them quite often there is an expectation of manual therapy and being on the phone it just completely takes it out of the equation. You don’t have to quite justify why you’re not doing the manual therapy quite as much because it’s just not an option.” [Physiotherapist] [47] Empowerment (+) “I was at home, I could relax, I could feel okay about what I was doing and I didn’t feel intimidated at all.” [Patient] [48] Motivation through support (+) “So it really helped to pick me up and actually having someone talk. Physio phoned up and spoke to me a few times, and that was really, really helpful, because it’s really encouraging that, ‘No, it’s all right keep moving, keep going.’” [Patient] [59] Access to health care (+) “I think the positive would be that I could do it at home, so I didn’t have to incorporate travel time and money for petrol, and trying to get there after work and all that type of stuff.” [Patient] [40] Societal awareness (+) “It is normal to experience back pain and it is often benign, which means that patients don’t have to restrict their activities. I sometimes wish that there was a more general understanding of back pain in society. This type of information could easily be shared through an application, I think.” [General practitioner] [56] Continuous care chain (+) “So that when they go away, and they think about it, that they have the opportunity to you know, reengage with the information if they haven’t taken it all on board at the time of the consultation.” [Physiotherapist] [33] Blended care (+) “A hybrid model would be awesome for people... maybe the first three weeks in person to really nail technique...then almost last three weeks via Telehealth so that they can learn to exercise in their own home environment.” [Physiotherapist] [44] Quality of care (+ and –) “You don’t necessarily need to be putting your hands on [to assess]...that might be 30 s worth...most of the other information we get about that kind of diagnosis and planning is with our eyes, and our ears, and our brains, which we still have over a computer.” [Physiotherapist] [44] “Yeah, some joint mobilities are a little tricky via the computer. Because, again, it is all about knowing and feeling the sensation and the amount of pressure. What amount of distraction and how much is too much.”” [Physiotherapist] [41] Patient–health care professional relationship (+ and –) “You had the time to really investigate what was motivating them or what their main issues were. Whereas I guess if you were more face-to-face and doing more of a traditional role you would be more focused on their range of movement and their strength...it is more about finding out more about them as a person and helping them to remain motivated to continue with the program. I think over the phone facilitated that to a certain degree.” [Physiotherapist] [47] “Humans are social creatures and you sort of lose that when everyone’s in their individual rooms online. Yes, you can still see them. Yes, you still engage with them, but it’s a different engagement.” [Physiotherapist] [44] Privacy and safety (–) “After reinstalling the app on my phone, I had to look through my old e-mails to find the login code, and it’s, of course, strange that if anyone else gets his hands on that email, they can see all my exercises and my private information.” [Patient] [43]	
	Adaptability			Flexibility (+) “Somehow, you want to prevent it from turning into some kind of assembly line work, and that the therapist no longer thinks about the kind of care that they provide.” [Physiotherapist] [30] Specificity (+) “Basically, I think it is a good app. However, the questions appear too frequent, too standard.” [Patient] [39] Suitability (+) “There is going to be a group both of patients and GPs who just don’t want to engage with that type of model. But I think that will be the case no matter what model is designed or developed.” [General practitioner] [54] Evolving intervention (+) “Renewing the exercises, for me it’s a good thing, because if you put a little bit of change, that makes it more enjoyable. From the moment you start a new exercise, it will stimulate you.” [Patient] [61]	
	Complexity			Usability factors (+) “What you often see in information provision in digital applications is that information is too complicated or too difficult to practically apply.” [Physiotherapist] [30] Health care professional management (+) “I think it’s a shame that the physiotherapist did not know how the program worked.” [Patient] [28]	
	Design quality and packaging			Variety and range of content and functionalities (+) “Well I suppose the variety. It wasn’t just you should be active. There were reasons behind and the self-awareness. I think it’s complete.” [Patient] [59] Persuasive design (+) “Options such as ticking off assignments and knowledge that the physiotherapist had insight in the progress were experienced by patients as ‘something that serves as a carrot.’” [Patient] [45] Modality (+) “I would very much like to stress that it should be an app. It’s just that it would really help because it is really tricky on the phone. It’s hard in the gym I want to look at the examples really quick and remind myself... an app would be better. You can use it offline.” [Patient] [53]	
	Cost			Reduced number of treatment sessions (+ and –) “I think it [e-Health] can be very cost-effective for health care, especially for jaw complaints. You can see your orofacial physiotherapist less often because you already have your tools with you. I think it’s a very good idea.” [Patient] [29] “I believe this intervention is good for everyone, but especially for the healthcare insurers.” [Patient] [46] Patient expenses (+ and –) “I think the positive would be that I could do it at home, so I didn’t have to incorporate travel time and money for petrol, and trying to get there after work and all that type of stuff.” [Patient] [40] “General practitioners generally felt that it should be funded by sources other than patients: ‘Ideally it should be...provided for free.’” [Patient] [54]	
**Outer setting**					
	Patient needs and resources			Personal traits of patients (–) “Sufficient Internet skills and self-discipline were described as prerequisite to use the web-based component.” [Patient] [28] Entertaining strategies (+) “It needs to be fun...like an adventure or detective game. For people like me, it would work.” [Patient] [61]	
	External policy and incentives			Acceptance by stakeholders (+) “You sometimes get this kind of pessimism from general practitioners. It’s not that they don’t want better interventions, it’s just that they’re sceptical that they will truly become a routine easily accessible part of practice.”” [General practitioner] [54] Health care guidelines (–) “It would be easier when there would be a national e-Health policy.” [Physiotherapist] [46] Privacy regulations (–) “We do have big confidentially chunk of potential[lity] issues. We cannot send information over an email without the patient’s permission; we cannot send any personalized data over an email.” [Physiotherapist] [41] External financial incentive (–) “According to physical therapists, this lack of financial incentive was seen as a potential barrier to use the proposed intervention in practice.” [Patient] [62]	
**Inner setting**					
	Networks and communications			Communication channels (+) “I think it comes down to the practicalities to be honest for a lot of these systems whether they succeed or fail, and that’s about taking time with the communication that was set up and getting the foundation in place to be effective.” [General practitioner] [54] Personal relationship (+) “The idea of handing a patient over to an anonymous group of people...I don’t see a great attraction.” [General practitioner] [54]	
	**Implementation climate**				
		Tension for change	Accessibility of health care (+) “My father is from a small mountain town where there is almost no mobile coverage... and we don’t even talk about the internet (laughs).” [Physiotherapist] [49] Need for trustworthy information (+) “GPs found that patients would have difficulty in discerning accurate content from inaccurate content.” [Patient] [56]		
	**Readiness for implementation**				
		Compatibility	Change of treatment routines (+ and –) “Required them to give me a lot more input, you know, describing what’s going on a little bit more, it will eliminate, I suppose, some of my normal go-to tactics.” [Physiotherapist] [48] “Once you’d done a couple, it was like—yeah, this is okay, it’s going to work. And we learned as we went.” [Physiotherapist] [44] Incompatibility with other initiatives and guidelines (–) and incompatibility with existing payment structures (–) “There’s all these other things that are happening in the background that will influence how general practitioners engage with a programme like this. Thinking about how this will fit into the regular work of a general practitioner will make a big difference, to whether it succeeds or fails.” [General practitioner] [54] Information incongruence (–) “There’s a possibility that...the way that they approach the problem is going to be a little bit different to mine...every now and then it’s some seemingly innocent or innocuous comment the patient turns over and then brings it back to you and you have to sort of spend time addressing that.” [General practitioner] [54]		
		Learning climate	Support from colleagues (+) “Support from colleagues and the absence of a national e-Health guideline or standard influenced the use of e-Exercise.” [Patient] [46] Professional autonomy (+) “I had the idea that I was in charge of the treatment.” [Physiotherapist] [45]		
	**Knowledge and beliefs about the intervention**				
		Available resources	Technology-related issues (–) “I didn’t have earphones so I didn’t quite understand this whole process. I think it was the second time that I’d used it. His receptionist was fabulous in coaching me through it and she set it up.” [Patient] [40] Time (+ and –) “We have more time to focus on therapy, as it is web-based so many small chats with patients are cut and therapy session is focused.” [Physiotherapist] [41] “The physiotherapist thought it was too much [time spent on the app during treatment]. However, I thought, well, you know, if it is necessary, it is necessary.” [Patient] [30] Physical space (+) “People just sort of popping in or out, or doors opening, and external noise going on, or tradies in the house next-door...that was probably a barrier.” [Patient] [44] Electronic health records “We need to start looking at developing and rolling out, you know, electronic records...more equipment and more investment...as a nation, we are probably just a little bit behind...particularly in the public system.” [Physiotherapist] [33]		
		Access to knowledge and information	Health care professionals’ training (+) “I think we [as physiotherapists] got a lot of information prior the trial so for me all the documents that we received actually allowed the process to be very routine and very kind of straight forward and I think obviously once you have done one or two sessions it really starts to become just quite mechanically because you know what you are doing and you know what your expectations are.” [Physiotherapist] [60] Access for patients (+) “Physiotherapists reported that to improve implementation in the future they would need to improve their own proficiency in using TRAK and allow patients time to explore TRAK before a consultation.” [Patient] [51] Instructions (+) “To have a bit more resources that you could offer patients... like a video that patients could see and understand what a telehealth session is, whereas I think telehealth has been mentioned in the news a lot and certainly general practitioners use it a lot but they tend to just use it as a phone call, which I think is very, very different to the way physios utilize it.” [Physiotherapist] [44]		
**Characteristics of individuals**					
	Knowledge and beliefs about the intervention			Health care professional acceptance (+ and –) “The way you will work and the way you will give information to the patients and counsel people. Changes are coming, I am sure of that.” [Physiotherapist] [29] “It would not suit me at all. I would have preferred to see someone in real life.” [General practitioner] [42] Health care professionals’ job satisfaction (+ and –) “It was easier on my body.” [Physiotherapist] [48]	
**Process**					
	**Engaging**				
		Opinion leaders	Peer opinion leaders (+) “General practitioners who were not familiar with relevant web-based information for low back pain patients expressed that it was not common to actively search for new material to present to their patients. Only if relevant material was presented to them, and preferably by a coworker who could vouch for the material, would they consider recommending it to their patients. Only if relevant material was presented to them, and preferably by a coworker who could vouch for the material, would they consider recommending it to their patients.” [Patient] [56]		
	**Executing**				
		Key stakeholders (health care professional)	Involvement (+) “I have used the research concepts to improve the telerehab that I do in the clinic...it was much more vigorous and a bit more standardised [than] what we did so I found it very satisfying and I think I have got more confidence.” [Physiotherapist] [60] Willingness to try (+) “When asked directly about their level of willingness to try telerehabilitation if introduced into their N/OPSC&MDS facility, almost all participants stated that ‘would certainly be willing to give it a go.’” [Patient] [52] Organizational uncertainties (–) “The argument will be, with the way that the HHS’s [hospital and health service districts] are, who does it and who pays for it?” [Physiotherapist] [52] Support team (+) “Participants considered the telerehabilitation technical support team as part of team providing therapy and they all expressed that they felt well supported by the entire team at all times.” [Patient] [50]		
			Justification and delegation (–) “I thought that they might feel a bit self-conscious being at the gym and chatting away, but most of them wholeheartedly just come and had no issue whatsoever with doing it, so that was good...but I had a couple of the gyms that did not enjoy the patients having their shoes off during the sessions so we had a number of those where either negotiate with the gym or they [patients] had to wear different shoes.” [Physiotherapist] [60]		

**^a^**CFIR: Consolidated Framework for Implementation Research.

#### Domain 1: Digital Health Service Characteristics

##### Innovation Source (High Reliability)

Commercially neutral digital health services may facilitate implementation according to health care professionals because logos of, for example, pharmaceutical companies could indicate economic instead of public health interests [42,56]. A link with an institution with a good image, such as a specialized hospital, may also be a facilitator to implementation, according to health care professionals, because it promotes trust [53].

##### Relative Advantage (High Reliability)

When patients or health care professionals experience a relative advantage of digital health services over usual care, this may facilitate implementation. Mentioned relative advantages were promoting *adherence* [28-30,33,43,49,53,61,62]*, self-management* [29-31,33,34,36,40,42-44,47,48,53,59]*, empowerment* [34,42,44,48,53,55]*, motivation through support* [28,31-33,37,38,43,53,59-61]*, access to health care* [29,34,36,38,40,41,44,45,47-50,52-54,56,60,62]*, creating societal awareness* [56] for specific health problems, and a *continuous care chain* [33,36,39,42,44,55,61]. The integration of digital health and therapy sessions *(blended care)* is described by patients and health care professionals as a facilitator because the digital health service can then be tailored to patient’s needs, complementary therapy can be offered, and self-efficacy can be enhanced [28-30,32,35,39-44,48,52,53,56,62]. However, there were also some concerns among health care professionals that *quality of care* may be reduced because, for example, physical examination may not be as thorough compared to usual face-to-face care [30,32-34,36,38-44,52,54,57]*.* On the contrary, some health care professionals believed that extra time and encouragement for the patient through a digital health service may result in better treatment outcomes. Advantages and disadvantages related to the *patient–health care professional relationship* were also experienced as both barriers and facilitators to implementation by patients and health care professionals [29-31,33,34,40,41,44,47,50,52,53,55]. Patients reported, for example, that when having health concerns, they prefer face-to-face reassurance over reassurance through a digital health service. Physiotherapists also had some concerns about creating a professional relationship if there are none or less face-to-face sessions. In contrast, they experienced that consulting via telephone forced them to focus on effective conversations, which allowed them to talk at a more personal level with patients. In addition, *privacy and safety* concerns may be barriers to implementation [36,40,42-44,52]. During the COVID-19 pandemic, safely providing health care from home was reported as a facilitator for implementation.

##### Adaptability (High Reliability)

Both health care professionals and patients agreed that adaptability of digital health services to fit the local context may be an important facilitator to implementation. Digital health services that are *flexible* to tailor to *specific* patient needs and *suitable* for various groups or subgroups of patients facilitate implementation [28-34, 39, 41, 42, 44-46, 48, 50, 52-54, 56, 57, 59, 61, 62]. Another facilitating determinant was an *evolving intervention* [43,56,61]. Use of a digital health service may increase if its content changes and information and features are continuously updated.

##### Complexity (High Reliability)

Complexity of digital health services that affect implementation is mostly linked to *usability*. Facilitating determinants concerning *usability* may be easy installation; easy to use; simple design and interface; simple navigation; visual support of text; and a not too wordy, manageable content [28-30,37,40,43-45,48,50,51,53,56,59,60]. Barriers concerning *usability* may be functional limitations of digital health services used in health care compared to those available on the commercial market. Another facilitating determinant to implementation was sufficient *health care professional management* for patients*,* such as updating relevant links and personal plans or the provision of technical aid by health care professionals to reduce complexity [28,53,55].

##### Design Quality and Packaging (High Reliability)

Experienced excellence in design quality and packaging of digital health services, such as *variety and range of content and functionalities* [29,30,32,35,37,45,53,59,61,62]*, persuasive design* [45,53,56]*,* and *modality* [33,34,40,45,53,61], may facilitate implementation according to both patients and health care professionals. Some mentioned functionalities are personal plans, exercise logs with speech notes as an alternative to text input, information modules with educational videos alongside written information, a progress dashboard with milestones, email or chat support, reminder tools, and feedback functions. An app was preferred over a website as modality, in particular, because of offline functionalities of an app.

##### Cost (High Reliability)

Costs associated with digital health services may be a barrier to implementation. Next to direct costs, a potential *reduced* *number of treatment sessions* [29,46,52,55,62] may both be a barrier and facilitator to implementation. Potential loss of income because of substitution of treatment sessions was experienced as a barrier by health care professionals. However, reducing treatment sessions may be a facilitator to some health care professionals because of efficiency, and offering innovative interventions attracts new patients, which is a financial incentive. Some health care professionals mentioned that *patient expenses* for digital health services may be a barrier to implementation [29,34,40,41,47,54]. In addition, digital health services may improve access to care for patients living in remote areas and may save them travel expenses, which was experienced as a facilitator to implementation.

#### Domain 2: Outer Setting

##### Patient Needs and Resources (High Reliability)

Needs of patients may influence the participation in digital health. *Personal traits of patients*, such as poor digital literacy [28,33,42,45,46,49,51,52], poor communication skills [34,41,47], higher age [36,41,42,44,45,56], lack of motivation [28,31,35,38,42,44,45,51,53,58,61], maladaptive illness perceptions [36,61], and feeling depressed [61], may be barriers to adherence or participation and therefore to implementation of digital health in primary care. Moreover, *entertaining strategies* for performing exercises, such as exercises in a video game, might improve engagement according to patients, which facilitates implementation [61].

##### External Policy and Incentives (High Reliability)

Broad acceptance of digital health by patients, health care professionals, and health service funders creates trust for health care professionals that implementation is worthwhile. Therefore, *acceptance by these* *stakeholders*, or even the demand by stakeholders such as patients, may be an important facilitator to implementation [29,31,32,44,54]. The absence of *health care guidelines* [44,46], standards, or protocols in using digital health and strict *privacy regulations* [41] may be barriers to implementation. Another barrier to health care professionals may be a lack of *external financial incentive* if the digital health intervention aims to substitute treatment sessions [62].

#### Domain 3: Inner Setting

##### Networks and Communications (High Reliability)

Effective, useful, and timely *channels of communication* between health care professionals involved in the use of a digital health intervention may be facilitators to implementation [39,52,54]. An example is the quality and quantity of communication between a general practitioner and a care support team that provided remotely delivered interventions in a multidisciplinary intervention. Another facilitator is some sort of *personal relationship* between health care professionals that are involved in using a digital health service [54].

##### Implementation Climate—Tension for Change (High Reliability)

Health care professionals and patients agreed that there is a need for change, which was a facilitator to implementation of digital health. Problems that create a tension for change are poor *accessibility to health care* [49,52,57] because of for example medical comorbidities, poor health literacy or inconvenient appointment times, large distance to health care service, high burden of health care on health care professionals, no availability of a (specialized) health care professional, and the *need for trustworthy information* [56]*.*

##### Implementation Climate—Compatibility (High Reliability)

Integrating digital health services into usual care requires *change of treatment routines,* which may be a barrier to implementation, specifically because of lack of knowledge and practice to adapt routines, lack of confidence, and resistance to change of health care professionals [29,30,39,44,46-48,51,52,54]. Positive experiences with integrating digital health services into usual care may lead to more acceptability and may overcome this barrier. Moreover, *incompatibility with other initiatives and guidelines* may be barriers to implementation [54]. There are many initiatives and guidelines for management of musculoskeletal conditions, and whenever these are incompatible with a digital health service, treatment routines may become complicated and confusing. In addition, *incompatibility with existing payment structures* may lead to inequity of care and was a barrier to implementation according to health care professionals [54]. Health care professionals mentioned that information incongruence could be another barrier to implementation [54]. Safety may be affected when patient advice and information, provided by health care professionals and via digital health services, are incongruent and as a consequence cause the health care professional to spend extra time and effort to deal with conflicting messages.

##### Implementation Climate—Learning Climate (High Reliability)

The extent to which health care professionals feel as essential, valued, and knowledgeable partners in the implementation process creates a better climate for implementation. Facilitators to implementation of digital health services may be *support from colleagues* and that the *professional autonomy* of health care professionals was maintained [45,46].

##### Readiness for Implementation—Available Resources (High Reliability)

Available resources, including the availability of suitable infrastructure, may facilitate the implementation of digital health. *Technology-related issues* may be a barrier to implementation [32,33,36,38,40,43,48-51,53,55,60]. Both patients and health care professionals mentioned several technology-related issues, including troubles with initially setting up or operating the technology, insufficient battery life, poor or no internet connection, poor video quality, and audio problems. Moreover, *time* may both be a barrier as well as a facilitator to implementation [28,30,33,41,45,46,51,52,56]. Some health care professionals perceived digital health services as time saving, whereas others perceived it as an additional burden. This issue involves the lack of time to familiarize with, set up, personalize, and use the technology as well as the time investment required from health care professionals to assist patients. In addition, the lack of a quiet *physical space* for health care professionals as well as patients specifically for telerehabilitation may be a barrier to implementation [33,44,52,61]. Moreover, the lack of *electronic health records* may be a barrier to implementation [33].

##### Readiness for Implementation—Access to Knowledge and Information (High Reliability)

Access of health care professionals and patients to knowledge and information about the use of digital health services may be an important determinant that influences implementation. A *health care professionals’ training* before using the digital health intervention may be a facilitator to implementation [41,44-47,52,60]. *Access for patients* to explore the digital health intervention before a consultation and clear *instructions* in the form of a manual, webinar, videos, or face-to-face support were facilitators to implementation [30,32,40,43,44,46,51].

#### Domain 4: Characteristics of Health Care Professionals (Knowledge and Beliefs About the Intervention: High Reliability)

Health care professionals’ acceptance of a digital health intervention may both be a facilitator and barrier [29,33,42,44-47,52,54,60,62]. Resistance to change of health care professionals may be a barrier to implementation, but if health care professionals trust that their efforts to embrace change will be worthwhile, this may facilitate implementation. Most health care professionals are open to digital health services, as long as they have appropriate training and time to familiarize with the intervention and its content. If experiences with a digital health intervention exceeds health care professionals’ expectations, this results in intrinsic motivation for the digital health intervention, which promotes implementation. The feeling of maintaining professional autonomy and confidence of health care professionals in being able to deliver the digital health intervention may also facilitate implementation. Concerns about patient information confidentiality, the belief that a digital health intervention will not be as good as face-to-face care, and providing digital health for conditions perceived as low priority may be barriers to implementation related to health care professional acceptance. Moreover, concerns that health care professionals’ job satisfaction may diminish may be a barrier to implementation [48,54,60]. However, if digital health services enable more contact with patients, this is experienced as a promotion of health care professionals’ satisfaction. Another contribution to satisfaction was that digital health services may lead to less physically demanding care compared to usual care, which all may facilitate implementation.

#### Domain 5: Process

##### Engaging—Opinion Leaders (Medium Reliability)

Peer opinion leaders exert influence through their representativeness and credibility. When new digital health services are presented to a health care professional by a coworker who vouches for it (*peer opinion leader*), this may facilitate implementation [56].

##### Engaging—Key Stakeholders (Health Care Professional; High Reliability)

*Involvement* of health care professionals in the implementation of digital health services is a facilitating determinant to implementation that promotes confidence in digital health services [60]. Furthermore, the willingness of health care professionals to try digital health services may facilitate implementation [52]. Organizational uncertainties among key stakeholders, such as questions like “Who does it?” and “Who pays for it?” may be barriers to implementation [54]. In addition, setting up a technical *support team* may lead to feelings of support by the health care professional, which may facilitate implementation [50].

##### Executing (Medium Reliability)

Executing the implementation of digital health services might require some *justification and delegation* to key involved stakeholders, such as gym staff [60]. This may be a barrier to implementation as, for example, content of the digital health intervention (eg, specific gym exercises) may not always be conventional.

## Discussion

### Principal Findings

In this systematic review, barriers and facilitators to the implementation of digital health services for people with musculoskeletal conditions in the primary health care setting were identified and synthesized according to the CFIR. Barriers and facilitators were identified within all 5 CFIR domains, and almost all constructs or subconstructs of the CFIR with synthesized barriers or facilitators had high reliability. Various stakeholders are involved in the implementation of digital health services for patients with musculoskeletal conditions in the primary care setting. The current determinant analysis provides a generic overview of barriers and facilitators that may be considered by stakeholders, such as digital health intervention developers, health care professionals, health care organizations, health policy makers, health care funders, and researchers, to design fitting implementation strategies [63]. As stakeholders mainly have influence on barriers and facilitators in specific CFIR domains, main results for stakeholders will be presented and discussed accordingly.

Identified barriers and facilitators that may especially be important for developers are from the domain “digital health service characteristics.” Facilitators within this domain include the flexibility of digital health services to tailor to specific patient needs, suitability for various subgroups, and high usability. Digital health service developers can consider these facilitators when developing and evaluating their product by using, for example, an eHealth framework, such as the Center for eHealth Research Roadmap [64]. An example of an existing digital health service that uses some of these facilitators is eHealth platform Physitrack, which was experienced by physiotherapists as user friendly, accessible, and helpful in providing personalized care [65,66]. Intervention design with nonoptimal usability was also identified as a barrier to implementation in other contexts, just as costs [14-17]. In this study, financial aspects, such as loss of income for health care providers because of potential substitution or patient expenses, were also shown to be important barriers to implementation for this specific context. Financial strategies to overcome these barriers when implementing digital health services for the context of patients with chronic illnesses living at home, such as changing the (patient) billing systems and fee structures, were suggested in previous research and may be relevant for developers to consider [67].

Identified barriers and facilitators that are especially important to health care professionals are from the domain “digital health service characteristics” and “outer setting.” A facilitator within the domain “digital health service characteristics” is the relative advantage of digital health over usual care, such as promoting adherence, self-management, empowerment, and access to health care. Important barriers are the concern that digital health services might negatively affect patient–health care professional relationship and quality of care, experienced additional burden of digital health services, and change of treatment routines. Existing workflow was also shown to be an important barrier in other contexts [16]. To use these facilitators and overcome these barriers, health care professionals might consider using previously developed implementation strategies used in another context, such as conducting educational meetings to train and educate colleague health professionals or conducting cyclical small tests of change [68]. Personal traits of patients, such as digital literacy, maladaptive illness perceptions, poor communication skills, and lack of confidence in the patient’s own physical ability, are barriers from the “outer setting.” An example of a previously developed tool for physiotherapists is the use of the Checklist Blended Physiotherapy [69]. This clinical decision aid to support the physiotherapist in the decision of whether a digital health service should be an integral part of physiotherapy treatment for an individual patient might be a strategy, which has yet to be evaluated.

Identified barriers and facilitators that are especially important to health policy makers are mostly from the domain “outer setting.” The lack of health care guidelines and lack of an external financial incentive were identified as barriers. The World Health Organization developed guideline recommendations on digital health services that can be used to develop guidelines for local contexts [9]. Changing reimbursement policies and clinician incentives are financial strategies that may are recommended to health policy makers [67]. Moreover, broad acceptance of digital health services by patients, health care professionals, and health service funders creates trust for health care professionals that implementation is worthwhile, which may facilitate implementation.

Identified barriers and facilitators that are especially relevant to health care organizations are mostly from the domain “inner setting.” Providing access to knowledge and information about the digital health intervention was found to be an important facilitator. In addition, an opinion leader and involvement of health care professionals facilitates implementation. Therefore, it is suggested that health care organizations consider implementation strategies, such as developing and distributing educational material as well as identifying and preparing champions, and inform local opinion leaders to develop stakeholder interrelationships [68]. Important barriers to overcome are technology-related issues and incompatibility with other initiatives, guidelines, and existing payment structures. Organizational uncertainties, such as questions like “Who does it?” and “Who pays for it?” are barriers to implementation that health care organizations must mainly overcome. To overcome these barriers, health care organizations are suggested to consider new sources of funding, involve executive boards, and try to form or join an innovation network [68].

Researchers can use the generic overview of barriers and facilitators of all domains to prioritize them for a local context, develop implementation strategies, test them, and systematically evaluate implementation outcomes. This is important because determinants are specific to the local context, and local contexts are ever changing [19].

Although several studies have identified barriers and facilitators to the implementation of digital health services in other settings than primary care or complex interventions in the primary care setting, this is the first systematic review of studies identifying and analyzing the facilitators and barriers of digital health services for people with musculoskeletal conditions in the primary health care setting. The results of this study are consistent with findings in other settings or the general health care setting [70]. Although the findings on the level of CFIR domains or subdomains are comparable to other contexts, the nuance in the description of the identified barriers and facilitators are mostly specific to primary care for patients with musculoskeletal conditions.

A strength of this systematic review is that all included articles had a mixed methods or qualitative design, and end-user perspectives of both patients and health care professionals were included, which led to a rich description of barriers and facilitators. However, it is important to note that many of the included studies did not follow a structured implementation process, and it was not possible to discuss whether implementation duration influenced the participants’ perspectives. Another strength is the use of the CFIR. Synthesizing according to the CFIR makes our findings easier comparable to other implementation studies and supports the use of common terminology in this field. Despite the careful execution of this study, there are some methodological considerations. The quality of the qualitative component was assessed by presenting stars. Cutoff values were determined by the authors; however, these cutoff values are arbitrary, which may have influenced the interpretation of the quality of included articles. In addition, a reliability rating was used to indicate confidence in the findings. While this approach took consistency and quality of the studies into account, we acknowledge that tools such as GRADE-CERQual were not used, which assesses confidence in findings from a more comprehensive perspective, considering factors such as coherence and adequacy. Incorporating GRADE-CERQual or similar methods in future research could enhance confidence in findings of a qualitative data synthesis [71]. The context of this review was digital health services, the primary care setting, and musculoskeletal conditions. People with musculoskeletal conditions are one of the largest patient groups in the primary health care setting. Although this patient group is very heterogenous, there are some transcendent key recommendations for patients with musculoskeletal conditions in primary health care, which makes the context sufficiently specific to inform relevant stakeholders [72]. Specific types of digital health services researched in the included articles were also very heterogenous. Therefore, it was not possible to specify barriers and facilitators to implementation for different types of digital health services. This should be considered when developing implementation strategies for specific digital health services. This systematic review provides a generic overview, and reliability was presented on the level of subconstructs and not on the level of individual determinants. Therefore, a prioritization of determinants should be carried out for the local context, as a first step in designing implementation strategies [19].

### Conclusions

This systematic review provides an extensive description of the barriers and facilitators to the implementation of digital health services for people with musculoskeletal conditions in the primary health care setting. The findings are based on the synthesis of 35 qualitative and mixed methods articles through the CFIR. Barriers and facilitators were identified across all 5 CFIR domains, and nearly all constructs or subconstructs of the CFIR with synthesized barriers or facilitators had high reliability. This suggests that the implementation process can be complex and requires implementation strategies across all CFIR domains. Stakeholders, such as digital health intervention developers, health care professionals, health care organizations, health policy makers, health care funders, and researchers, can consider the identified barriers and facilitators to design tailored implementation strategies after a prioritization has been carried out in their local context.

## Appendix

Multimedia Appendix 1 Search strategy.

Multimedia Appendix 2 Preferred Reporting Items for Systematic Review and Meta-Analysis (PRISMA) checklist.

## Abbreviations

CFIR: Consolidated Framework for Implementation Research
MMAT: Mixed Methods Appraisal Tool

## Data Availability

The data sets generated and analyzed during this study are available from the corresponding author on reasonable request.
