# Supplementary material for: Barriers and Facilitators to the Implementation of Digital Health Services for People With Musculoskeletal Conditions in the Primary Health Care Setting: Systematic Review
Source: J Med Internet Res. 2024 Aug 27;26:e49868. doi: 10.2196/49868 (PMC11387918; doi:10.2196/49868)
Supplement: Multimedia Appendix 1 [file jmir_v26i1e49868_app1.docx]

| Appendix 1. Search strategy | | | |
| --- | --- | --- | --- |
| Database | **Date** | **Search strategy** | **Hits** |
| PUBMED |  |  |  |
|  | 04-03-2024 | ((Physical Therapy Specialty[MeSH Terms]) OR (Physical Therapists[MeSH Terms]) OR (Physical and Rehabilitation Medicine[MeSH Terms]) OR (Public Health Nursing[MeSH Terms]) OR (Rehabilitation Nursing[MeSH Terms]) OR (Orthopedic Nursing[MeSH Terms]) OR (Home Health Nursing[MeSH Terms]) OR (primary care nursing[MeSH Terms]) OR (Nurses, Community Health[MeSH Terms]) OR (Exercise Therapy[MeSH Terms]) OR (Occupational Therapists[MeSH Terms]) OR (General Practitioners[MeSH Terms]) OR (Physical Therap*[Title/Abstract]) OR (physiotherap*[Title/Abstract]) OR (Public Health Nurs*[Title/Abstract]) OR (Rehabilitation Nurs*[Title/Abstract]) OR (Orthopedic Nurs*[Title/Abstract]) OR (Home Health Nurs*[Title/Abstract]) OR (primary care nurs*[Title/Abstract]) OR (community health nurs*[Title/Abstract]) OR (exercise therap*[Title/Abstract]) OR (Occupational Therap*[Title/Abstract]) OR (General Practitioners[Title/Abstract])) OR ((Primary Health Care[MeSH Terms]) OR (Allied Health Occupations[MeSH Terms]) OR (Allied health personnel[MeSH Terms]) OR (Primary Health Care[Title/Abstract]) OR (allied health[Title/Abstract]) OR (Primary care[Title/Abstract])) AND ((implementation science[MeSH Terms]) OR (implementation science[Title/Abstract]) OR (implement*[Title/Abstract]) OR (implementing[Title/Abstract]) OR (implementation process[Title/Abstract]) OR (health plan implementation[MeSH Terms]) OR (health plan implementation[Title/Abstract]) OR (adopt*[Title/Abstract]) OR (routin*[Title/Abstract]) OR (integrat*[Title/Abstract]) OR (uptake[Title/Abstract]) OR (implementation[Title/Abstract]) OR (diffusion of innovations[Title/Abstract]) OR (dissemination[Title/Abstract]) OR (knowledge to action*[Title/Abstract]) OR (knowledge transfer[Title/Abstract]) OR (knowledge translation[Title/Abstract]) OR (research to practice[Title/Abstract]) OR (research utilization[Title/Abstract]) OR (scale up[Title/Abstract]) OR (process evaluation[Title/Abstract]) OR (Barrier*[Title/Abstract]) OR (Facilitate*[Title/Abstract]) OR (Usage[Title/Abstract])) AND ((telerehabilitation[MeSH Terms]) OR (telemedicine[MeSH Terms]) OR (telerehabilitation[Title/Abstract]) OR (telemedicine[Title/Abstract]) OR (telehealth[Title/Abstract]) OR (mhealth[Title/Abstract]) OR (mobile health[Title/Abstract]) OR (Remote Rehabilitation[Title/Abstract]) OR (internet[Title/Abstract]) OR (website[Title/Abstract]) OR (world wide web[Title/Abstract]) OR (Web based[Title/Abstract]) OR (internet based[Title/Abstract]) OR (ehealth[Title/Abstract]) OR (blended[Title/Abstract]) OR (smart phone[Title/Abstract])) AND ((Musculoskeletal System[MeSH Terms]) OR (musculoskeletal system[Title/Abstract]) OR (Musculoskeletal Abnormalities[MeSH Terms]) OR (Musculoskeletal Abnormalities[Title/Abstract]) OR (musculoskeletal pain[MeSH Terms]) OR (Musculoskeletal Pain[Title/Abstract]) OR (Musculoskeletal Diseases[MeSH Terms]) OR (Musculoskeletal Diseases[Title/Abstract]) OR (Orthopedic Disorders[Title/Abstract]) OR (knee[MeSH Terms]) OR (hip[MeSH Terms]) OR (low back[MeSH Terms]) OR (shoulder[MeSH Terms]) OR (neck[MeSH Terms]) OR (Hand[MeSH Terms]) OR (Elbow[MeSH Terms]) OR (Foot[MeSH Terms]) OR (Spine[MeSH Terms]) OR (knee[Title/Abstract]) OR (hip[Title/Abstract]) OR (low back[Title/Abstract]) OR (shoulder[Title/Abstract]) OR (neck[Title/Abstract]) OR (Hand[Title/Abstract]) OR (Elbow[Title/Abstract]) OR (Foot[Title/Abstract]) OR (Spine[Title/Abstract])) | 494 |
| EMBASE |  |  |  |
|  | 04-03-2024 | 'primary health care'/exp OR 'physiotherapy'/exp OR 'home physiotherapy'/exp OR 'home rehabilitation'/exp OR 'primary medical care'/exp OR 'community health nursing'/exp OR 'community based rehabilitation'/exp OR 'rehabilitation nursing'/exp OR 'orthopedic nursing'/exp OR 'health practitioner'/exp OR 'manual therapist'/exp OR 'occupational therapist'/exp OR 'paramedical profession'/exp OR 'kinesiotherapy'/exp OR 'primary medical care':ab,ti OR 'primary health care':ab,ti OR 'paramedical profession':ab,ti OR physiotherapy:ab,ti OR physiotherapist:ab,ti OR 'home physiotherapy':ab,ti OR 'home rehabilitation':ab,ti OR 'community health nursing':ab,ti OR 'community based rehabilitation':ab,ti OR 'rehabilitation nursing':ab,ti OR 'orthopedic nursing':ab,ti OR 'health practitioner':ab,ti OR 'manual therapist':ab,ti OR 'occupational therapist':ab,ti OR kinesiotherapy:ab,ti OR 'general practitioner'/exp OR 'general practitioner':ab,ti AND 'implementation science'/exp OR 'health care planning'/exp OR 'implementation science':ab,ti OR 'health care planning':ab,ti OR implement*:ab,ti OR adopt*:ab,ti OR routin*:ab,ti OR integrat*:ab,ti OR uptake:ab,ti OR dissemination:ab,ti OR 'knowledge to action*':ab,ti OR 'knowledge transfer':ab,ti OR 'knowledge translation':ab,ti OR 'research to practice':ab,ti OR 'research utilization':ab,ti OR 'scale up':ab,ti OR 'process evaluation':ab,ti OR facilitator:ab,ti OR barrier:ab,ti OR usage:ab,ti AND 'telerehabilitation'/exp OR 'teletherapy'/exp OR 'telehealth'/exp OR 'mobile application'/exp OR 'mobile health application'/exp OR 'Internet'/exp OR 'web-based intervention'/exp OR 'smartphone'/exp OR telerehabilitation:ab,ti OR teletherapy:ab,ti OR telehealth:ab,ti OR 'mobile health application':ab,ti OR 'mobile health':ab,ti OR mhealth:ab,ti OR internet:ab,ti OR website:ab,ti OR 'web-based intervention':ab,ti AND 'musculoskeletal disease'/exp OR 'musculoskeletal pain'/exp OR 'musculoskeletal injury'/exp OR 'tendon disease'/exp OR 'ligament disease'/exp OR 'bone disease'/exp OR 'arthropathy'/exp OR 'contracture'/exp OR 'chondropathy'/exp OR 'musculoskeletal stiffness'/exp OR 'musculoskeletal system malformation'/exp OR 'musculoskeletal system inflammation'/exp OR 'ligament and tendon injury'/exp OR 'ligament injury'/exp OR 'tendon injury'/exp OR 'bone injury'/exp OR 'cartilage injury'/exp OR 'joint injury'/exp OR 'muscle injury'/exp OR 'spine injury'/exp OR 'sprain'/exp OR 'musculoskeletal disease':ab,ti OR 'musculoskeletal pain':ab,ti OR 'musculoskeletal injury':ab,ti OR 'tendon disease':ab,ti OR 'tendon injury':ab,ti OR 'ligament disease':ab,ti OR 'ligament injury':ab,ti OR 'muscle disease':ab,ti OR 'muscle injury':ab,ti OR 'bone injury':ab,ti OR 'bone disease':ab,ti OR arthropathy:ab,ti OR contracture:ab,ti OR chondropathy:ab,ti OR 'musculoskeletal stiffness':ab,ti OR 'musculoskeletal system malformation':ab,ti OR 'musculoskeletal system':ab,ti OR 'cartilage injury':ab,ti OR 'joint injury':ab,ti OR sprain:ab,ti OR 'low back pain'/exp OR 'knee'/exp OR 'knee disease'/exp OR 'hip'/exp OR 'hip disease'/exp OR 'shoulder'/exp OR 'shoulder disease'/exp OR 'neck'/exp OR 'neck pain'/exp OR 'cervical spine'/exp OR 'hand'/exp OR 'hand disease'/exp OR 'foot disease'/exp OR 'foot'/exp OR 'elbow'/exp OR 'elbow disease'/exp OR 'low back pain':ab,ti OR knee:ab,ti OR 'knee disease':ab,ti OR hip:ab,ti OR 'hip disease':ab,ti OR shoulder:ab,ti OR 'shoulder disease':ab,ti OR neck:ab,ti OR 'neck pain':ab,ti OR 'cervical spine':ab,ti OR hand:ab,ti OR 'hand disease':ab,ti OR 'foot disease':ab,ti OR foot:ab,ti OR elbow:ab,ti OR 'elbow disease':ab,ti AND [embase]/lim NOT ([embase]/lim AND [medline]/lim) | 308 |
| CINAHL |  |  |  |
|  | 04-03-2024 | ( (MH ( general practitioner or family doctor or primary care physician or gp ) OR AB ( general practitioner or family doctor or primary care physician or gp ) OR TI ( general practitioner or family doctor or primary care physician or gp ) ) OR (MH "Physical Therapy+") OR (MH "Physical Therapy Practice, Evidence-Based") OR (MH "Physical Therapy Practice, Research-Based") OR (MH "Physiotherapy Evidence Database") OR (MH "Primary Health Care") OR (MH "Home Rehabilitation+") OR (MH "Community Health Nursing") OR (MH "Community Health Workers") OR (MH "Community Health Services+") OR (MH "Home Rehabilitation+") OR (MH "Nursing Home Personnel") OR (MH "Orthopedic Nursing") OR (MH "Occupational Therapists") OR (MH "Allied Health Professions+") OR (MH "Allied Health Personnel+") OR TI ( 'primary medical care' OR 'primary health care' OR 'paramedical profession' OR physiotherapy OR physiotherapist OR 'home physiotherapy' OR 'home rehabilitation' OR 'community health nursing' OR 'community based rehabilitation' OR 'rehabilitation nursing' OR 'orthopedic nursing' OR 'health practitioner' OR 'manual therapist' OR 'occupational therapist' OR kinesiotherapy ) OR AB ( primary medical care OR primary health care OR paramedical profession OR physiotherapy OR physiotherapist OR home physiotherapy OR home rehabilitation OR community health nursing OR community based rehabilitation OR rehabilitation nursing OR orthopedic nursing OR health practitioner OR manual therapist OR occupational therapist OR kinesiotherapy ) ) AND ( (MH "Program Implementation") OR (MH "Implementation Science") OR (MH "Program Development+") OR TI ( 'implementation science' OR 'health care planning' OR implement* OR adopt* OR routin* OR integrat* OR uptake OR dissemination OR 'knowledge to action*' OR 'knowledge transfer' OR 'knowledge translation' OR 'research to practice' OR 'research utilization' OR 'scale up' OR 'process evaluation' OR facilitator OR barrier OR usage ) OR AB ( 'implementation science' OR 'health care planning' OR implement* OR adopt* OR routin* OR integrat* OR uptake OR dissemination OR 'knowledge to action*' OR 'knowledge transfer' OR 'knowledge translation' OR 'research to practice' OR 'research utilization' OR 'scale up' OR 'process evaluation' OR facilitator OR barrier OR usage ) ) AND ( (MH "Telerehabilitation") OR (MH "Telehealth+") OR (MH "Mobile Applications") OR (MH "World Wide Web Applications+") OR (MH "Cellular Phone+") OR (MH "Smartphone") OR (MH "Internet+") OR (MH "Internet-Based Intervention") OR (MH "World Wide Web Applications+") OR (MH "World Wide Web+") OR TI ( telerehabilitation OR teletherapy OR telehealth OR 'mobile health application' OR 'mobile health' OR mhealth OR internet OR website OR 'web-based intervention' OR ‘internet-based intervention’ OR ‘world wide web’ ) OR AB ( telerehabilitation OR teletherapy OR telehealth OR 'mobile health application' OR 'mobile health' OR mhealth OR internet OR website OR 'web-based intervention' OR ‘internet-based intervention’ OR ‘world wide web’ ) ) AND ( (TI ( foot or feet or lower limb or lower extremities or elbow or hand or upper extremity or upper limb or arm or neck or cervical spine or cervical or shoulder or shoulder joint or glenohumeral or hip or knee OR low back pain or lumbar pain or lumbar spine pain or non specific low back pain or chronic low back pain ) AND AB ( foot or feet or lower limb or lower extremities or elbow or hand or upper extremity or upper limb or arm or neck or cervical spine or cervical or shoulder or shoulder joint or glenohumeral or hip or knee OR low back pain or lumbar pain or lumbar spine pain or non specific low back pain or chronic low back pain ) AND MH ( foot or feet or lower limb or lower extremities or elbow or hand or upper extremity or upper limb or arm or neck or cervical spine or cervical or shoulder or shoulder joint or glenohumeral or hip or knee OR low back pain or lumbar pain or lumbar spine pain or non specific low back pain or chronic low back pain ) ) OR (MH "Musculoskeletal Abnormalities+") OR (MH "Musculoskeletal System+") OR (MH "Musculoskeletal Diseases+") OR (MH "Diagnosis, Musculoskeletal+") OR TI ( 'musculoskeletal disease' OR 'musculoskeletal pain' OR 'musculoskeletal injury' OR 'tendon disease' OR 'tendon injury' OR 'ligament disease' OR 'ligament injury' OR 'muscle disease' OR 'muscle injury' OR 'bone injury' OR 'bone disease' OR arthropathy OR contracture OR chondropathy OR 'musculoskeletal stiffness' OR 'musculoskeletal system malformation' OR 'musculoskeletal system' OR 'cartilage injury' OR 'joint injury' OR sprain ) OR AB ( 'musculoskeletal disease' OR 'musculoskeletal pain' OR 'musculoskeletal injury' OR 'tendon disease' OR 'tendon injury' OR 'ligament disease' OR 'ligament injury' OR 'muscle disease' OR 'muscle injury' OR 'bone injury' OR 'bone disease' OR arthropathy OR contracture OR chondropathy OR 'musculoskeletal stiffness' OR 'musculoskeletal system malformation' OR 'musculoskeletal system' OR 'cartilage injury' OR 'joint injury' OR sprain ) ) | 133 |
